# The Druggists' General Receipt Book

**Published:** 1853-01

**Authors:** 


*The Druggists' General Receipt Book.* Philadelphia, Lindsay & Blakiston, 1853.

A very useful collection of formulæ which the druggist will know how to value. Veterinary prescriptions, nostrums, perfumery, cosmetics, condiments, sick food, the common chemicals are all to be found here.

